# Two salamander species respond differently to timber harvests in a managed New England forest

**DOI:** 10.7717/peerj.7604

**Published:** 2019-08-30

**Authors:** Angus Mossman, Max R. Lambert, Mark S. Ashton, Jessica Wikle, Marlyse C. Duguid

**Affiliations:** 1 Department of Ecology and Evolutionary Biology, Yale University, New Haven, CT, United States of America; 2 Department of Environmental Science, Policy and Management, University of California, Berkeley, Berkeley, CA, United States of America; 3 School of Forestry and Environmental Studies, Yale University, New Haven, CT, United States of America

**Keywords:** Amphibian, Chronosequence, Wildlife habitat, Coarse woody debris, Logging, Second growth forest, Red-backed salamander, Eastern newt

## Abstract

**Background:**

Managing forests for timber while protecting wildlife habitat is of increasing concern. Amphibians may be particularly sensitive to forest management practices due to their unique biology; however, it is not clear how different species respond to timber harvest practices—particularly over longer time scales.

**Methods:**

Here we report on the differential responses of two salamander species—the eastern red-backed salamander (*Plethodon cinereus* Green) and the eastern newt (*Notophthalmus viridescens* Rafinesque)—to forest harvesting, by examining communities across a 25-year chronosequence of regenerating shelterwood harvests.

**Results:**

Populations of both species were lowest immediately after harvest, but increased at substantially different rates. Red-backed salamander populations were highest in 20–25 year-old shelterwoods—significantly higher than in mature, unharvested, control (100–120 year old) stands. Eastern newt populations, however, were greatest in unharvested control stands and still had not recovered to population levels found in mature stands in the 25 years since harvest. Red-backed salamander abundances were strongly tied to stand age as well as abundance of decayed coarse woody debris, suggesting that timber harvests influence some wildlife species by affecting a suite of interacting habitat variables that change over time. In contrast, newt abundances were not directly related to stand age but were more related to downed wood and vegetation characteristics. Our results highlight markedly variable responses by two common salamander species to forest harvesting—species with markedly different life histories and reproductive patterns—and that time since harvest may be useful in predicting abundance.

## Introduction

Managing forests to promote wildlife and maintain ecosystem resilience is increasingly important due to myriad anthropogenic environmental impacts including climate change, habitat fragmentation, and widespread biodiversity loss (Rands et al., 2010). Timber harvesting directly influences non-target species through ground disturbance and changes in stand structure (Horn, Babb & Gunther, 1983; Halpern & Spies, 1995; Lindenmayer, Margules & Botkin, 2000; Sullivan & Sullivan, 2001; Duguid & Ashton, 2013; Duguid et al., 2016). Yet, more work is needed in northeastern United States forests detailing how different species within the same functional groups (e.g., ground-dwelling species) respond to forest management. Most management guidelines focus on responses of more charismatic fauna (e.g., songbirds and game species; Gibbons, 1988; Goodale et al., 2009; Duguid et al., 2016). Prior efforts have emphasized that we need to better understand how poorly-studied groups of wildlife respond to forest management and studies are needed to help managers set goals that incorporate the implications of various silvicultural treatments on wildlife (Lindenmayer, Franklin & Fischer, 2006).

Amphibians are an important component of forests in the northeastern United States—the biomass of salamanders alone may be twice that of resident mammals and therefore play an important role in food webs and nutrient cycling (Burton & Likens, 1975; Walker et al., 2018). Many amphibian species have experienced population declines over the past 50 years, in large part due to anthropogenic activities (Houlahan et al., 2000; Stuart et al., 2004; Grant et al., 2016). Among those activities, forest practices may have a disproportionate impact on amphibians due to their sensitivity to changes in micro-habitat (Welsh & Droege, 2001; Knapp et al., 2003; Morneault et al., 2004; Semlitsch et al., 2009; Otto, Roloff & Thames, 2014; Warren & Ashton, 2014; O’Donnell, Thompson III & Semlitsch, 2015; Greenberg et al., 2016). That sensitivity highlights the importance of examining amphibians as a conservation priority in forest management. While forest harvesting has been shown to decrease some amphibian populations, research has mostly focused on how populations respond to complete canopy removal (primarily clearcuts; DeMaynadier & Hunter Jr, 1995; Stoddard & Hayes, 2005; Karraker & Welsh Jr, 2006). In the northeastern United States, more recent research has addressed responses to other forest harvest treatments (Knapp et al., 2003; Semlitsch et al., 2009; Otto, Kroll & McKenny, 2013; O’Donnell, Thompson III & Semlitsch, 2015; Greenberg et al., 2016), but generally over relatively short time frames post-harvest (often < 5 years). True clearcuts are rare in northeast hardwood forests, with many foresters opting for shelterwood harvests to facilitate regeneration in hardwood forests. Shelterwood harvesting opens up enough of the canopy to foster increased regeneration of shade intolerant species such as oak (*Quercus* spp. L.) and hickory (*Carya* spp. Nutt.), while retaining enough overstory to provide shelter, as well as a seed source, for regeneration (Leak, Yamasaki & Holleran, 2014; Duguid et al., 2016; Ashton & Kelty, 2018). The presence of the residual structure can lead to effects on species that differ from clearcutting (Duguid et al., 2016). Residual trees may be especially important for herpetofauna and other fossorial species because they may act as mini-refugia within the disturbed landscape (Rittenhouse et al., 2008; Rosenvald & Lõhmus, 2008; LeGros, Steinberg & Lesbarrères, 2017). Hence, amphibian responses to shelterwood harvest particularly warrant further study, especially over long time scales that enable evaluating amphibian response through stand regeneration.

Regeneration harvests, such as shelterwoods, drastically alter forest structure by removing the majority of canopy trees. This initially converts the stand into shrubby, early successional habitat, but these stands are dynamic and quickly move into stem-exclusion stage (*sensu* Oliver & Larson, 1996) within the first twenty years (Oliver & Larson, 1996; Duguid et al., 2016; Ashton & Kelty, 2018). Vegetation characteristics of these stands (e.g., basal area, sapling density) as well as amount of CWD directly contribute to the suitability of amphibian habitat (Greenberg, 2001; Matthews et al., 2010). These forest stand attributes are commonly and easily measured and tracked by forest managers and are often correlated with other important environmental variables (e.g., soil moisture, temperature), and so are useful for characterizing environmental differences likely experienced by salamanders (Gálhidy et al., 2006; Latif & Blackburn, 2010). Most studies to date, have examined responses of amphibian populations to harvest shortly after harvest (1–3 years; e.g., Pough et al., 1987; Harpole & Haas, 1999; Bartman et al., 2001; Cantrell et al., 2013), though a few studies have returned after stand regeneration (15 to 25 years; DeGraaf & Yamasaki, 2002; Ford et al., 2002). Some work has also compared salamander assemblages in recent clear cuts to older mature stands unharvested over 50 years (Petranka, Eldridge & Haley, 1993). However, what is lacking is an understanding of the dynamics of these populations following harvest as the forest moves through stand development (Ash, 1997; Moorman, Russell & Greenberg, 2011).

We designed this study to begin addressing this knowledge gap. Here we survey two dominant salamander species, the eastern red-backed salamander (*Plethodon cinereus* Green) and the eastern newt (*Notophthalmus viridescens* Rafinesque; terrestrial red eft juvenile stage) across a chronosequence of 14 shelterwood harvests from 3–25 years old, and compare survey results with those from mature (100–120 year old) reference stands. Our approach, based on a chronosequence, offers a unique opportunity to infer salamander responses over a relatively long time frame and provide a contrast to salamander responses in mature, unharvested stands. These mature, second-growth stands comprise mostly mixed hardwood with some conifers that have not been cut in many decades—long past the amount of time that salamanders are typically thought to need to recover from timber harvests (Petranka, Eldridge & Haley, 1993; Ash, 1997).

The purpose of our study was to consider the response of two common salamander species (red-backed salamander and eastern newt) to shelterwood harvests. We compared relative abundances with respect to age and forest development, utilizing only commonly available and easily gathered forest metrics for managed forests. This study sheds light on the temporal dynamics of amphibian populations following forest management in southern New England.

## Materials & Methods

### Study area

Our study was located at the Yale-Myers Forest (YMF), a 3213-ha research and demonstration forest located in northeastern Connecticut (41°57′N, 72°07′W). The forest sits on the ancestral lands of the Nipmuk people, is classified as central hardwood-hemlock-pine (Westveld, 1956) and is currently composed primarily of second-growth hardwood, developing since the removal of old-field pine between 1900–1920 that became established in the mid-nineteenth century (∼1850) after agricultural abandonment by European colonial farmers (Meyer & Plusnin, 1945; Ashton et al., 2015). The climate is temperate (mean summer temperature 21 °C, winter −2 °C) with an annual mean rainfall of 123 cm (NOAA, 1981–2010 Climate Normals). As part of an active timber management regime, over the past twenty-five years the forest has undergone regeneration harvesting, primarily in the form of irregular shelterwoods. Irregular refers to the temporal organization in these shelterwoods. Irregular shelterwoods retain a greater proportion of residual canopy trees as reserve trees than traditional uniform shelterwood systems, increasing vertical structure as well as microhabitat and species diversity and resulting in a more heterogeneous forest structure in the regenerated stand (Raymond et al., 2009; Ashton & Kelty, 2018). At Yale-Myers, reserves left in these irregular shelterwoods can be either single tree or small group, creating irregularity over both space and time. The average size of one of these shelterwood harvests is about 8 ha, but they can range from 2 to 20 ha (Goodale et al., 2009).

### Site selection

**Figure 1:**
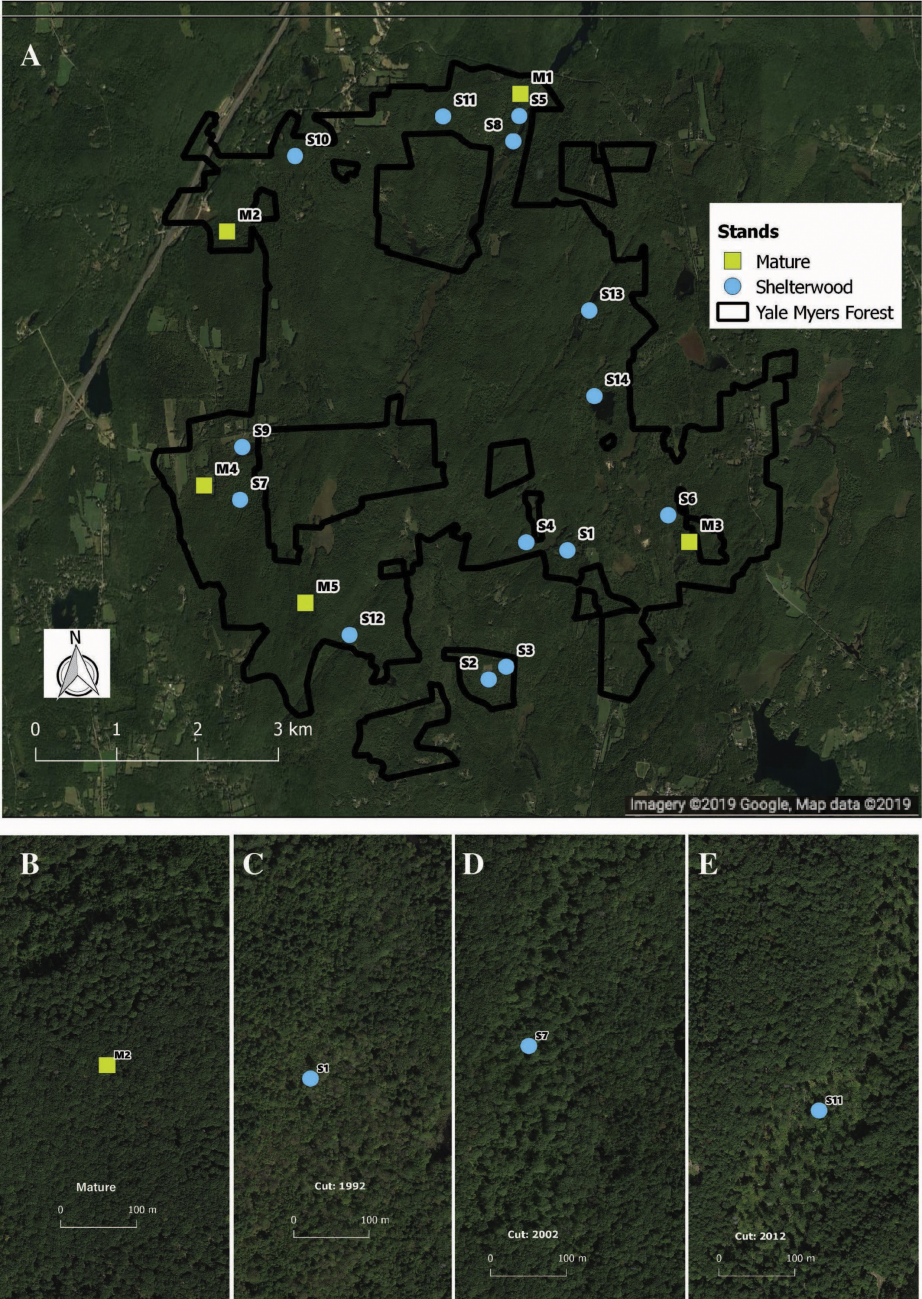
Location and example of sampled stands. Distribution (A) of shelterwood and mature stands across the Yale-Myers Forest in northeastern Connecticut, USA. Below are a representative mature stand (B), 26-year-old sheltewood (C), 16-year-old shelterwood (D), and 6-year-old shelterwood (E). Maps were produced in qgis, Map data ©2019 Google.

We capitalized on an existing chronosequence designed to assess long-term vegetation dynamics following shelterwood harvesting. This chronosequence was specifically designed across similar sites and has previously been used to examine bird population dynamics (Goodale et al., 2009; Duguid et al., 2016). While chronosequences cannot take the place of long-term studies, they can provide useful inferences about species responses to environmental change and are tractable for the characteristics of our study system (Johnson & Miyanishi, 2008; Walker et al., 2010). To avoid potential confounding effects of having stands entered repeatedly for harvest, we selected stands that had been harvested only once. In total, we sampled 19 stands—14 shelterwoods harvested between 1992 and 2014, as well as five mature second-growth (100–120 years) stands. The mature stands had similar tree species composition, soil type, and original land-use history as the shelterwood stands pre-harvest (Fig. 1). Pre-harvest, dominant overstory species included black birch (*Betula lenta* L.), sugar maple (*Acer saccharum* Marshall), red maple (*Acer rubrum* L.), red oak (*Quercus rubra* L.), white oak (*Quercus alba* L.), eastern hemlock (*Tsuga canadensis* Carrière), hickory, and white pine (*Pinus strobus* L.). The dominant regenerating tree species in the shelterwoods include black birch, red maple, and striped maple (*Acer pensylvanicum* L.) saplings, with dominant shrub species of mountain laurel (*Kalmia latifolia* L.), witch hazel (*Hamamelis virginiana* L.), and raspberry (*Rubus* spp*.*). All shelterwoods and mature forests are located in uplands with coarse-loamy mesic Dystrudept soils formed from till in the Charlton-Chatfield, Paxton-Montauk, Brimfield-Brookfield, and Woodbridge soil series (Web Soil Survey, 2019). For a general comparison of all stands surveyed see Table S1.

### Experimental design

**Figure 2:**
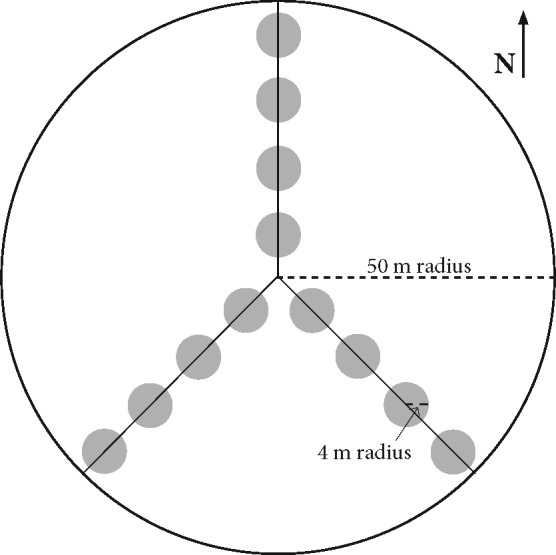
Experimental Design Layout. Sample plots utilized for salamander, Coarse Woody Debris (CWD), and vegetation surveys. 50 m transects radiated out from a center point to the North, Southeast, and Southwest and there were four evenly-spaced plots of 4-meter radius along each transect used for CWD, salamander, and sapling surveys. Overstory tree data was collected in the larger 50 m radius plot.

At each of the 19 stands we sampled 12 four-meter radius (∼50 m^2^) plots. Plots were arranged along three transects radiating out from a randomly selected center point to the north, southeast, and southwest (Fig. 2). The center point was randomly selected from a restricted region that was at least 75 m from the stand boundary so as to avoid the edge effect of adjacent stand conditions (Goodale et al., 2009).

We conducted a single salamander survey between June 19th and July 9th 2017, sampling each stand once, thus constraining the sampling period to minimize seasonal climactic variability. To further minimize climactic influences on detectability, we restricted sampling to between 8:45 and 16:15 when temperatures were between 15.6 °C–28.3 °C. We did not sample during or immediately following precipitation events, under complete cloud-cover, or during high-wind. Respective mean monthly temperature and rainfall for the region during sampling in June and July were: 20.6 °C, 9.17 cm; 22.8 °C, 16.69 cm (NOAA monthly climate records). In each of the 228 50 m^2^ plots (12 plots in each of the 19 stands), we counted and identified every individual of all amphibian species found on or beneath any piece of CWD. We left all remaining substrate in a stand undisturbed. We searched completely each piece of substrate that originated within the plot, even if part of a piece extended beyond the edge of the plot. When possible, we flipped over CWD of all decay classes and scanned the space beneath before lightly disturbing the ground layer to search for any hiding animals. For wood substrate of decay class 4 or greater, as per Woodall & Monleon (2008), we systematically dismantled the wood to reveal any amphibians. This included lifting moss or other debris from the substrate’s surface. Where applicable, we repeated the same procedure for stumps. We acknowledge this was destructive sampling, but these 12 plots represent a small proportion of each stand. The shelterwood harvest stands in this study ranged from 4.23–17.1 hectares in size, so even in our smallest stand sampling area represents less than 1% of total stand area. Nonetheless, we replaced all substrate as accurately as possible to its pre-survey position. We minimized handling animals, and when we encountered salamanders in substrate we gently placed them under nearby substrate after counting. Our surveys were in accordance with Yale IACUC protocol 2015-110681.

In each stand we measured coarse woody debris (CWD) at six of the 12 plots, alternating which pair of plots received CWD measurements between each consecutive transect and stand (e.g., 1st and 3rd plots on north transect, 2nd and 4th on southeast transect). We measured all CWD ≥ five cm diameter that maintained contact with the ground: we measured the length of each piece of wood within the fixed area plot and diameter at both ends. If debris had multiple axes, we measured only branches that fit the above requirements. We treated stumps similarly except only the height of stump and diameter at small end (if ≥5 cm) were measured, providing conservative surface area and volume measurements. We also recorded decay class per Woodall & Monleon (2008). All CWD was measured during the survey interval.

For habitat structure we used the center point of each stand as the center for a 50-meter radius overstory plot (0.785 ha). For each tree in the plot we recorded species and diameter at breast height (dbh = 1.37 m) for all trees with dbh >15 cm. For tree regeneration we measured the diameter at root collar, and height (from ground to terminal bud) of seedlings (trees <1.3 m) in all 12 subplots. At the first and third measurement plot on each transect, we used a plot size of 2.82 m radius (25 m^2^) and recorded species, dbh, and height for all saplings (>1.3 m with dbh ≤15 cm).

### Statistical analysis

We used R (Version 1.0.143; R Core Team, 2018) for all analyses to explore which variables might influence abundances of the two salamander species. We scaled all variables to the stand level for analysis. We selected variables to use in models by examining a correlation matrix of all habitat variables to detect collinearity (Table S2). We removed redundant variables and those that had correlation coefficients >0.6. We chose the following variables to use in our models: (i) years since harvest; (ii) volume of CWD; (iii) number of saplings per hectare; and iv) overstory basal area (BA).

To examine the relationship of the abundance of both red-backed salamanders (RBS) and eastern newts (EN) with forest harvesting variables we built negative binomial generalized linear models (GLMs) using the glm.nb() function in the “MASS” package (Venables & Ripley, 2002). We note that the term “abundance” refers to an abundance index as we were unable to account for detection probabilities and directly assess abundances. We calculated standardized coefficients using the “lm.beta” package (Behrendt, 2014) and adjusted r-squared values for each GLM using the rsq() function in the “rsq” package (Zhang, 2018). We used the vif() function in the “car” package to test for variance inflation and confirm that collinearity among predictor variables was not confounding our results (all values <3 ; Fox & Weisberg, 2018). We designated statistical significance as *α* ≤ 0.05, and marginal significance as 0.05 < *α* ≤ 0.10.

## Results

**Figure 3:**
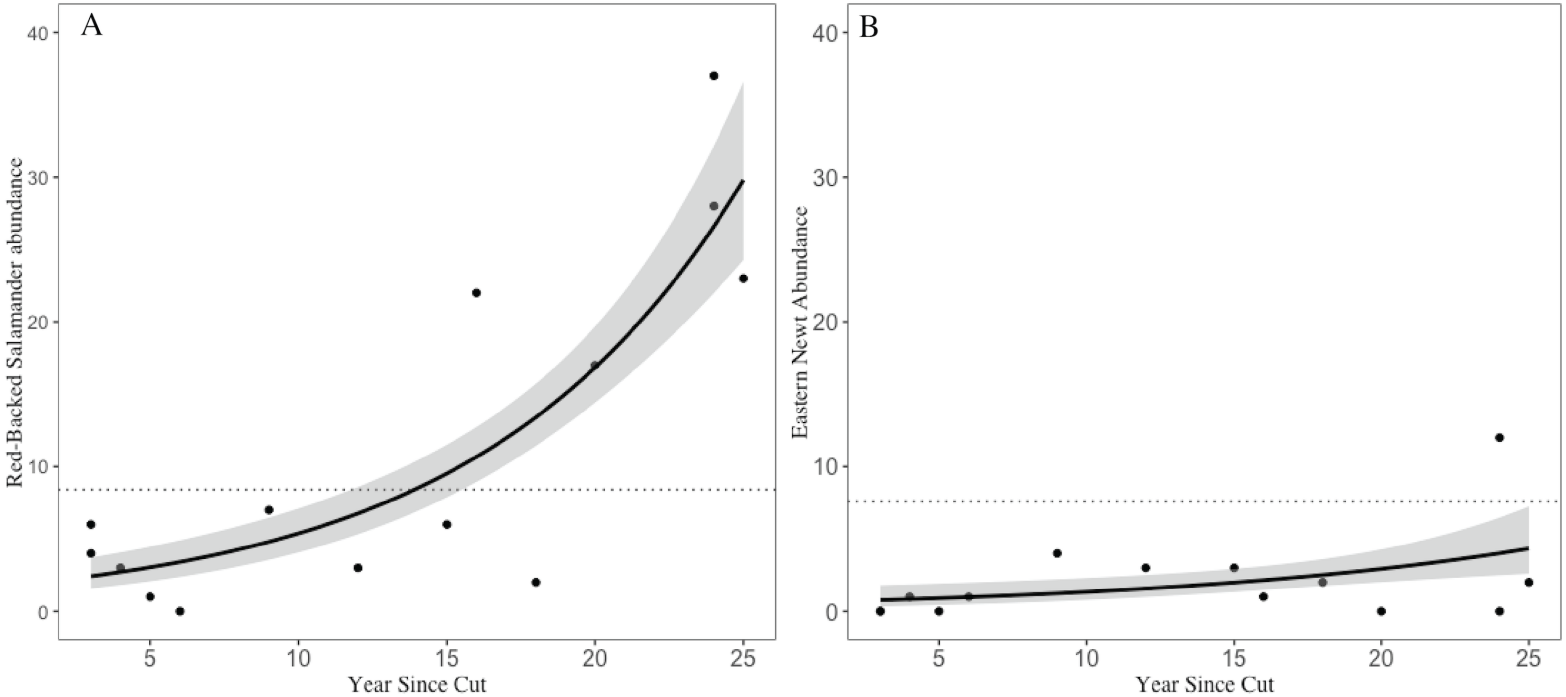
Trends in salamander abundance through the chronosequence. The relationship of (A) red-backed salamander abundance and (B) eastern newt abundance with number of years since stand harvesting. The dotted line represents the mean value for uncut mature reference stands. 95% confidence intervals shown with gray shading.

Across the 14 shelterwood stands we encountered 159 RBSs ($\bar {x}=11.36$, SE = 3.15) and 29 ENs ($\bar {x}=2.07$, SE = 0.84). In the five mature reference stands we encountered 42 RBSs ($\bar {x}=8.4$, SE = 3.14) and 38 ENs ($\bar {x}=7.6$, SE = 2.01). The GLM for RBS had an adjusted *R*^2^ of 0.76, significant variables included years since harvest, volume of CWD, and overstory basal area. For ENs, the GLM had an adjusted *R*^2^ of 0.90 with volume of CWD, and overstory basal area as significant, but year since harvest and number of saplings were marginally significant (Table 1). Years since harvest had a positive relationship with both RBS and EN abundances, but with a different magnitude of effects; RBS abundances in older shelterwoods (>15 years) were 2–4 times higher than the average abundance observed in mature stands whereas EN abundances in shelterwoods were ∼50–80% lower than abundances in mature reference and still increasing (Fig. 3).

**Table 1:** Results of generalized linear models. Negative binomial generalized linear models with both red-backed salamander and eastern newt abundance as response variables against time since harvest and habitat variables. Variables are shown with both their estimated unstandardized coefficients as well as standardized coefficients (SE).

	*β*	**est**	**se**	*z*	*p*	
**Red-backed Salamanders**						
(Intercept)	0.000	0.089	0.982	0.091	0.927	
Year	0.102	0.146	0.027	5.490	0.000	***
Volume of CWD	0.039	0.006	0.002	2.716	0.007	**
Saplings	−0.011	0.000	0.000	−0.578	0.563	
Basal Area	−0.041	−0.128	0.041	−3.141	0.002	**
**Eastern Newts**						
(Intercept)	0.000	−2.699	1.840	−1.467	0.142	
Year	0.224	0.086	0.046	1.865	0.062	.
Volume of CWD	0.246	0.010	0.004	2.774	0.006	**
Saplings	−0.274	0.000	0.000	−1.914	0.056	.
Basal Area	0.184	0.153	0.062	2.481	0.013	*

## Discussion

Timber harvests impact forest habitats. Our study offers evidence that shelterwood harvests and subsequent regeneration influence two salamander species in different ways. Both RBSs and ENs seemed to have low numbers in recently harvested stands, but the two species differed greatly in both their rate and magnitude of recovery. Specifically, we observed that RBSs not only recovered quickly, but abundances in older shelterwoods (15-25 years since harvest) greatly exceeded those in the mature reference forest stands. EN abundances, however, were still substantially lower than mature forest populations even 25 years after harvest. It is important to note that this study was not designed to provide baseline abundance data, but to compare across age and structure in a managed forest. Although imperfect, we base our definition of recovery here on the population numbers found in the mature stands with the assumption these adequately represent the available habitat as discussed below. The species-specific responses we identified are important. Forest managers may view salamanders as a single group, but RBSs and ENs belong to different families with different life history traits. If we do not understand how land use differentially impacts some of the most common species, it will be challenging to manage for more rare or threatened species in mixed-use landscapes.

Our results suggest some species of amphibians may recover in regenerating forests faster than the literature suggests. Previous studies propose that it takes time for amphibian populations to return to pre-harvest levels in a stand following canopy removal, but a review by Moorman, Russell & Greenberg (2011) found that the details are widely disputed and have ranged from 20 to 100 years. By assessing shelterwood cuts, our study differs from much of the previous work that focused only on clearcuts (Petranka, Eldridge & Haley, 1993; Ash, 1997) and/or wide time intervals (Petranka, Eldridge & Haley, 1993; Herbeck & Larsen, 1999; DeGraaf & Yamasaki, 2002; Ford et al., 2002). We suggest that the relatively rapid recovery we observed for red-backed salamanders is influenced by the nature of irregular shelterwoods in contrast to clearcuts. Since irregular shelterwoods retain legacy trees in the regenerating stand, reserve canopy trees may be able to function as essential refugia for salamanders during harvesting due to their ability to help maintain soil moisture (Rosenvald & Lõhmus, 2008; Leak, Yamasaki & Holleran, 2014). These reserves combined with relatively small patchy harvest units and irregular edges may contribute to increased connectivity for dispersal and subsequent re-establishment in the stand given RBSs have dispersal distances that are generally less than 10 m (Liebgold, Brodie III & Cabe, 2011). Further, irregular shelterwoods generally leave more woody debris on the ground than clearcuts based on market conditions and wood utilization (Ashton & Kelty, 2018). This additional CWD once sufficiently decayed creates more habitat for these dispersing amphibians to utilize.

Some research has found that shelterwoods generally reduce *Plethodon* salamander abundances in the four years following cutting but have apparently little effect on EN abundance in this time frame (O’Donnell, Thompson III & Semlitsch, 2015; Greenberg et al., 2016), which agrees with our results for RBSs but less so for ENs. The different response patterns between the two species may reflect different life history traits. Newts of the eastern United States are unique among other local amphibians in possessing a tri-phasic life cycle with aquatic larvae, terrestrial juveniles (efts), and aquatic adults (Pough, 1974; Roe & Grayson, 2008). All ENs we identified were in their terrestrial eft stage which is marked by high dispersal ability and a tolerance for low-moisture environments (Pough, 1974; Gibbs, 1998). RBSs, however, have very limited dispersal ability, are tied to narrow ranges of soil moisture and pH, and are fully terrestrial throughout their lives (Heatwole, 1962; Wyman & Hawksley-Lescault, 1987; Gibbs, 1998; Grover, 1998). As a result, most individual RBSs in a stand are likely residents. In spite of low immigration by RBSs, with enough time, the progression of forest succession supports a high growth rate of RBS populations. ENs, on the other hand, are transient in this life phase and so do not necessarily represent resident populations within a stand, but more likely the preferences of the species in this life stage. EN efts can travel up to 100 m in a single night (Roe & Grayson, 2008) and can have home ranges of up to several hundred m^2^ (Healy, 1975) indicating that efts can readily traverse through shelterwood habitats in their 3-7 year terrestrial phase before becoming adults. Neither of these species of salamanders exhibit the annual migration typical of many other salamander species in this region (e.g., Ambystomatid). Together, our findings emphasize the importance of taking species identity and natural history into account when considering management consequences on short-term and long-term scales.

To our knowledge, little work has investigated the relationship between salamander abundance and stand regeneration over medium-long time periods. Our work begins to fill this gap by capitalizing upon a shelterwood chronosequence spanning 25 years post-cut at almost annual intervals. Past studies have emphasized the importance of CWD in influencing amphibian distributions and population levels (McKenny, Keeton & Donovan, 2006; Strojny & Hunter, 2009), however, a literature review by Otto, Kroll & McKenny (2013) on amphibian responses to downed wood in managed forests suggests that future research incorporate the temporal dynamics of CWD as well as how the characteristics and distribution of CWD in a stand influence amphibian populations. These studies point to a noticeable gap in how amphibian populations and their habitats change over time following forest management. Our findings suggest that time since harvest, an easily measurable proxy variable, can be useful in predicting salamander responses to silvicultural treatments. This work also shows that, although CWD diminishes as stands regenerate, cut stands with higher CWD densities also host more red-backed salamanders when controlling for stand age.

Shelterwood treatments affect a suite of environmental variables which can in turn influence abundance and distribution of forest-dwelling amphibians in different ways. Such variables include changes in canopy cover and incident light, soil moisture, vegetation species composition, volume of CWD, and vertical structure (Leak, Yamasaki & Holleran, 2014). Our results reveal RBS abundance is most intimately connected with stand age, while ENs are more influenced by CWD. Together, this indicates that while stand age is a useful predictor alone for RBS, ENs respond more strongly to other stand attributes which should be taken into consideration along with stand age.

Our results showed RBS abundance was roughly three times higher in 25-year old shelterwood harvests than in control stands. One explanation is that because RBSs rely on CWD for habitat structure (Grover, 1998), the increased CWD immediately following forest harvesting, given time to decay, can create habitat that may be scarce in many mature forest stands today. Because CWD abundance is negatively correlated with stand age (Fig. S1A), a univariate model suggests RBS abundance is negatively correlated with CWD abundance (Fig. S1B). However, our model incorporates both stand age and CWD abundance and suggests that, controlling for stand age, RBS abundances are positively correlated with CWD abundance (Fig. S1C). In this study we use mature, second-growth forests as our control stands. Our stands were between 100–120 years old, an age class associated with low levels of CWD (Jenkins & Parker, 1997), which may partly explain the lower abundance of RBSs found in these stands. However, since true old-growth forests are extremely rare in eastern deciduous forests, we feel that these are representative controls for understanding how regional forest management affects salamanders, although future work should assess true old-growth if possible.

## Conclusions

Our results offer novel insight into the temporal dynamics of upland salamander abundance in managed forests. We show that patterns for some species can be gauged from a single variable (time since harvest) and a short sampling window. While longer and more detailed surveys may provide more data, forest managers can more reasonably incorporate this level of monitoring into their post-harvest evaluations. Incorporating amphibian conservation goals into forest management practices will require considering the particular needs of individual species. Forest managers should identify the target species and utilize information on ecology, phenology, and ontogeny when deciding the timing and scale of harvesting operations. Given RBS abundances appear to eventually benefit from regenerated cuts whereas EN abundances appear to remain highest in older mature stands, the results here suggest that creating a patchwork mosaic of stands of different ages could improve the distribution of appropriate habitat for multiple species across a forested landscape. Our results suggest that managers monitoring post-harvest effects would benefit from considering both habitat structure and stand age. Future research should incorporate medium-long term (5–30 years post-harvest) monitoring/comparisons to include a broader and more continuous range of time since harvest, including pre-treatment data. Studies that focus on responses to harvest as a function of species traits—such as seasonal migrations, longevity, aquatic versus terrestrial breeding—and taxonomy are particularly important, as are studies that more closely evaluate species’ responses to harvest across seasons and which also account for variation in soil properties across sites and as succession proceeds.

##  Supplemental Information

10.7717/peerj.7604/supp-1 Table S1 Stand characteristics for all surveyed stands. Comparison of measured variables in shelterwood and mature stands. Age represents years since harvest. For CWD, Decay stands for mean decay class, Vol, for estimate of total volume, SA for surface area. Overstory data: density stands for stems per hectare and BA=basal area (m^2^/ha). Vegetation and Stand Size data were not available for mature stands.

10.7717/peerj.7604/supp-2 Table S2 Correlation matrix of habitat variables. Year: number of years since stand harvest, Pieces CWD: pieces of coarse woody debris (CWD) per stand (number/ha), Decay: average decay class of CWD (on a 1–5 scale), Volume CWD: volume of CWD per stand (m^3^/ha), SA CWD: surface area of CWD per stand (m^2^/ha), Trees: abundance of trees with dbh >15 cm per stand (number/ha), BA: basal area of trees with dbh >15 cm per stand (m^2^/ha), Saplings: sapling abundance per stand (number/ha).

10.7717/peerj.7604/supp-3 Figure S1 The relationship between abundance of red-backed salamanders and course woody debris (CWD) CWD is negatively correlated with age (A). While salamander abundance overall is negatively correlated with CWD (B), when you control for the age:CWD relationship salamander abundance is positively correlated with CWD (C) because of the CWD to age correlation. Functionally, more CWD correlates with more salamanders but only within the context of a stand’s age.

10.7717/peerj.7604/supp-4 Supplemental Information 1 Dataset of amphibian abundances and habitat measurements This dataset consists of the variables: (A) Stand_Name: harvest name given to the stand by foresters; (B) Red_Backed_Salamander: abundance of red-backed salamanders per stand; (C) Eastern_Newt: abundance of eastern newts per stand; (D) Total_Abundance_CWD: total salamander abundance observed; (E) Harvest_Year and (F) Year_Since_Cut: correlated measures of stand age; (G) pieces_of_wood: # pieces CWD; (H) Average_Wood_Decay_Class_per_Stand: average of the decay class; (I) Volume_m3_per_ha: volume of CWD in cubic meters per hectare; (J) Surace_Area_m2_per_ha: surface area of CWD in square meters per hectare; (K) Number_Overstory_Trees: number of stems measured in the 50 m radius plots; (L) Overstory_basal_area: basal area in square meters per hectare; (M) number_sapling_per_ha: count of saplings; (N) sapling height in centimeters.

10.7717/peerj.7604/supp-5 Supplemental Information 2 R code file The code that was used to analyze the dataset.

## Additional Information and Declarations

### Data Availability

The following information was supplied regarding data availability: The raw data is available as a Supplemental File and the code is available as an R file.
