# Supplementary material for: Two salamander species respond differently to timber harvests in a managed New England forest
Source: PeerJ. 2019 Aug 30;7:e7604. doi: 10.7717/peerj.7604 (PMC6718153; doi:10.7717/peerj.7604)
Supplement: Table S1 — Comparison of measured variables in shelterwood and mature stands. Age represents years since harvest. For CWD, Decay stands for mean decay class, Vol, for estimate of total volume, SA for surface area. Overstory data: density stands for stems per hectare and BA=basal area (m2/ha). Vegetation and Stand Size data were not available for mature stands. [file peerj-07-7604-s001.docx]

|  | Stand | |  | Coarse Woody Debris | | |  | Overstory | | |
| --- | --- | --- | --- | --- | --- | --- | --- | --- | --- | --- |
|  | Age | Size (ha) |  | Decay | Vol (m^3^/ha) | SA (m^2^/ha) |  | Density (stems/ha) | BA (m2/ha) | Saplings (stems/ha) |
| S1 | 3 | 10 |  | 2.333 | 312.4 | 5145.4 |  | 10 | 2.32 | 13200 |
| S2 | 3 | 4.98 |  | 2.539 | 203.7 | 3493.5 |  | 34 | 5.54 | 5600 |
| S3 | 4 | 10.6 |  | 2.756 | 277.3 | 5299.5 |  | 42 | 6.25 | 16267 |
| S4 | 5 | 4.43 |  | 3.009 | 160.8 | 3225.2 |  | 76 | 10.18 | 7400 |
| S5 | 6 | 11.6 |  | 3.156 | 162.3 | 2987.1 |  | 52 | 8.43 | 4067 |
| S6 | 9 | 5.26 |  | 3.313 | 281.6 | 4143 |  | 156 | 12.46 | 6200 |
| S7 | 12 | 8.41 |  | 3.381 | 327.3 | 4647.1 |  | 57 | 8.04 | 5933 |
| S8 | 15 | 4.7 |  | 3.831 | 149.8 | 2510 |  | 51 | 9.07 | 5933 |
| S9 | 16 | 4.23 |  | 3.621 | 222.8 | 4133.6 |  | 71 | 4.9 | 7333 |
| S10 | 18 | 8.41 |  | 3.632 | 172.4 | 1907.1 |  | 79 | 16.33 | 7333 |
| S11 | 20 | 15.3 |  | 3.429 | 76 | 1404.6 |  | 88 | 3.3 | 5733 |
| S12 | 24 | 12.2 |  | 3.667 | 190.8 | 2136.5 |  | 98 | 11 | 2733 |
| S13 | 24 | 17.1 |  | 3.574 | 122.2 | 2191.6 |  | 62 | 5 | 6133 |
| S14 | 25 | 16.4 |  | 3.553 | 116.3 | 1901.1 |  | 183 | 8.15 | 4067 |
| M1 | Mature | – |  | 3.46 | 96.3 | 2276.8 |  | – | – | – |
| M2 | Mature | – |  | 3.208 | 68.4 | 1594.2 |  | – | – | – |
| M3 | Mature | – |  | 3.545 | 37.1 | 983.8 |  | – | – | – |
| M4 | Mature | – |  | 3.462 | 133.7 | 1977.6 |  | – | – | – |
| M5 | Mature | – |  | 3.395 | 64.8 | 1089.6 |  | – | – | – |
