# Supplementary material for: Two salamander species respond differently to timber harvests in a managed New England forest
Source: PeerJ. 2019 Aug 30;7:e7604. doi: 10.7717/peerj.7604 (PMC6718153; doi:10.7717/peerj.7604)
Supplement: Table S2 — Year: number of years since stand harvest, Pieces CWD: pieces of coarse woody debris (CWD) per stand (number/ha), Decay: average decay class of CWD (on a 1–5 scale), Volume CWD: volume of CWD per stand (m3/ha), SA CWD: surface area of CWD per stand (m2/ha), Trees: abundance of trees with dbh >15 cm per stand (number/ha), BA: basal area of trees with dbh >15 cm per stand (m2/ha), Saplings: sapling abundance per stand (number/ha). [file peerj-07-7604-s002.docx]

|  | Pieces CWD | Decay | Volume CWD | SA CWD | Trees | BA | Saplings | Sapling height |
| --- | --- | --- | --- | --- | --- | --- | --- | --- |
| Year | -0.84 | 0.82 | -0.57 | -0.74 | 0.54 | 0.15 | -0.54 | -0.42 |
| Pieces CWD |  | -0.80 | 0.67 | 0.86 | -0.50 | -0.44 | 0.69 | 0.37 |
| Decay |  |  | -0.48 | -0.63 | 0.50 | 0.43 | -0.60 | -0.58 |
| Volume CWD |  |  |  | 0.91 | -0.30 | -0.03 | 0.52 | 0.30 |
| SA CWD |  |  |  |  | -0.41 | -0.28 | 0.69 | 0.30 |
| Trees |  |  |  |  |  | 0.43 | -0.48 | -0.15 |
| BA |  |  |  |  |  |  | -0.30 | -0.26 |
| Saplings |  |  |  |  |  |  |  | 0.28 |
