# Supplementary figures and images for: Two salamander species respond differently to timber harvests in a managed New England forest
Source: PeerJ. 2019 Aug 30;7:e7604. doi: 10.7717/peerj.7604 (PMC6718153; doi:10.7717/peerj.7604)

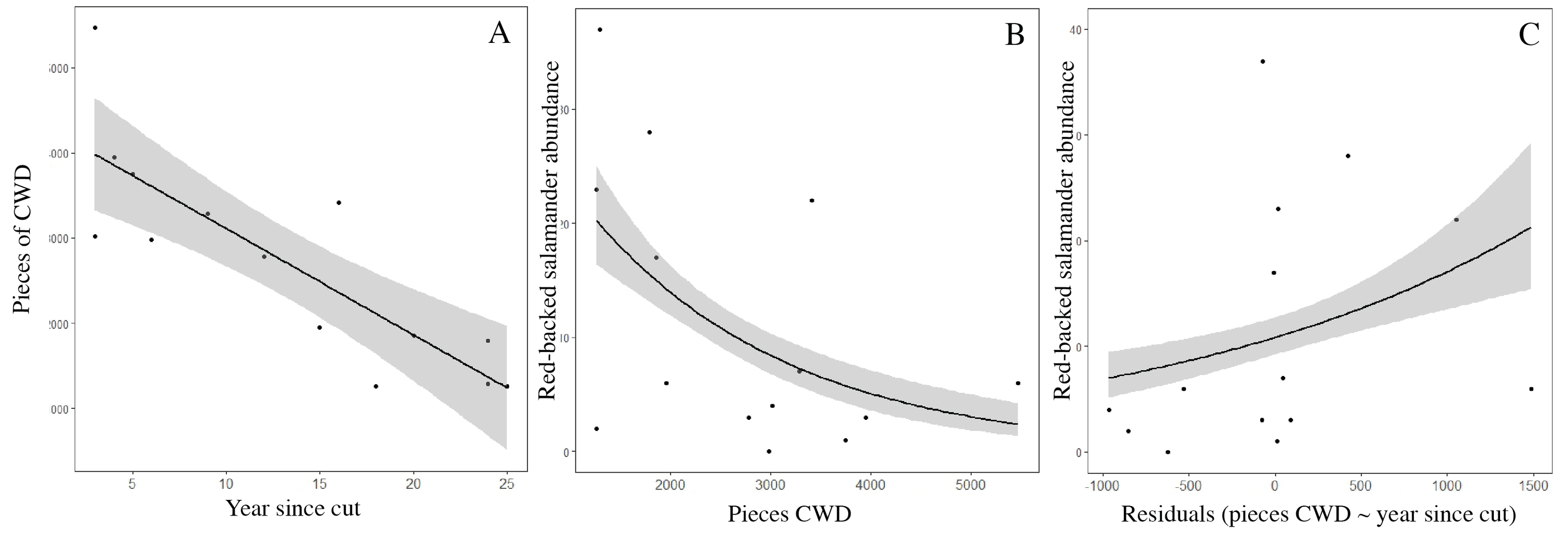

Supplement: Figure S1 — CWD is negatively correlated with age (A). While salamander abundance overall is negatively correlated with CWD (B), when you control for the age:CWD relationship salamander abundance is positively correlated with CWD (C) because of the CWD to age correlation. Functionally, more CWD correlates with more salamanders but only within the context of a stand’s age. [file peerj-07-7604-s003.png]
